# Expression of Locally Produced Adipokines and Their Receptors during Different Physiological and Reproductive Stages in the Bovine Corpus Luteum

**DOI:** 10.3390/ani13111782

**Published:** 2023-05-27

**Authors:** Granit Thaqi, Bajram Berisha, Michael W. Pfaffl

**Affiliations:** 1 Chair of Animal Physiology and Immunology, TUM School of Life Sciences, Technical University of Munich, 85354 Weihenstephan, Germany; bajram.berisha@uni-pr.edu (B.B.); michael.pfaffl@tum.de (M.W.P.); 2 Department of Animal Biotechnology, Faculty of Agriculture and Veterinary, University of Prishtina, 10000 Prishtina, Kosovo

**Keywords:** ovary function, corpus luteum, adipokines, gene expression, cattle reproduction

## Abstract

**Simple Summary:**

Adipokines are cytokines secreted by adipose tissue that play a crucial role in several biological processes, including energy balance, food intake, immune status, and reproduction. Research has shown that there is a close correlation between energy balance, metabolism, and reproductive function in mammals. Among these factors, ovarian physiology has been found to have its own local regulation. In this study, we aimed to gain insight into the local production of novel adipokines and their receptors in the corpus luteum of cycling and pregnant cows. We examined corpora lutea at various stages in cycling and pregnant cows to investigate the involvement of locally produced adipokines and their receptors in ovarian physiology. Our findings reveal significant differences in the expression levels of adipokines throughout the estrous cycle and pregnancy, indicating their potential role in corpus luteum lysis and/or resumption of cyclicity.

**Abstract:**

This study aimed to determine the gene expression of different local novel adipokines, such as vaspin, adiponectin, visfatin, and resistin, and their known receptors, namely, heat shock 70 protein 5, adiponectin receptor 1, and adiponectin receptor 2, in the bovine corpus luteum (CL) during different phases of the estrous cycle (on days 1–2, 3–4, 5–7, 8–12, 13–18, >18) and pregnancy (at months 1–2, 3–4, 5–7, >7). The mRNA expression was measured by reverse transcription polymerase chain reaction (RT-qPCR). The mRNA expression levels were normalized to the geometric mean of all three constantly expressed reference genes (cyclophilin A, ubiquitin, ubiquitin C). Our findings suggest that adipokines are expressed and present in all investigated groups, and are specifically up- or downregulated during the estrus cycle and during pregnancy. Vaspin and adiponectin levels were upregulated in the middle and late cycle stages. Resistin was abundant during the CL regression stage and in the first months of pregnancy. The specific expression of adipokine receptors indicates their involvement in the local mechanisms that regulate CL function. Further investigations are required to elucidate the regulative mechanisms underlying the different local effects of adipokines on the ovarian physiology of cows.

## 1. Introduction

The female reproductive system in domestic ruminants is regulated by a complex system of hormones produced by the hypothalamus, pituitary gland, and ovaries. This interaction of protein hormones (e.g., GnRH, LH, and FSH) and steroid hormones (e.g., estrogen and progesterone) results in ovarian cyclicity in females, which, in consequence, leads to fertilization, pregnancy, and parturition [1,2]. This series of events in ruminants is mainly controlled by the dominance of estrogen produced by ovarian follicles and progesterone produced by the corpus luteum (CL) [3,4].

No other organs in the body, when compared to the ovaries, are subject to dramatic changes in function and structure in such short intervals. These changes include cell proliferation, differentiation, and transformation, resulting in an ovulatory follicle, and hence, the formation, function, and regression of the CL. Nevertheless, cow fertility is much more complex than the interactions between these well-known reproductive hormones. Fertility is also tightly related to other components, such as locally secreted mediators, including growth factors, peptides, cytokines, steroids, prostaglandins, and many more [5,6,7,8,9]. Moreover, a family of cytokines derived from adipose tissue, called adipokines, affects the function of other tissues in an autocrine and paracrine fashion and whole-body homeostasis in an endocrine manner [10]. During the last decade, it has been shown that the adipose tissue is increasingly involved in regulating the energy balance, food intake, ovarian physiology, and immune status in domestic animals [11,12,13]. The adipose tissue is involved in the hormone production, regulation, and release of these adipokines, its own class of hormones that includes adiponectin, vaspin, visfatin, resistin, chemerin, leptin, and apelin [14,15,16]. Since the discovery of adipokines in 1994, thousands of studies on adipokines have been published, the majority of which are related to metabolic disorders in humans [17,18]. Their presence and expression have been documented in tissues derived from organs such as the brain, stomach, kidneys, pancreas, and liver [19,20]. They play important roles in multiple biological processes, such as insulin sensitivity regulation, food intake, and inflammation [21]. Their role in energy metabolism and maintaining metabolic homeostasis is currently widely accepted [10,22].

More recently, the involvement of adipokines in the regulation of fertility and the development of some reproductive disorders has been suggested [17,23,24]. Data concerning the role of adiponectin and leptin, the two most studied adipokines in mammals, are consistent, and confirm their effects on the reproductive axis in humans, pigs, mice, and cows [18]. In recent years, interest has grown in some novel adipokine candidates, e.g., chemerin, visfatin, resistin, and apelin, which are strongly associated with obesity and insulin resistance, mainly in humans [25,26]. These novel adipokines could thus represent metabolic sensors that regulate reproductive functions according to changes in energy balance. Vaspin (visceral adipose tissue-derived serine protease inhibitor), an adipokine that was discovered in 2005, is associated with the development of insulin resistance, obesity, and inflammation. A significantly higher level of vaspin was observed in obese patients [27]. Adiponectin is a protein hormone produced by the AdipoQ gene that modulates several metabolic processes, including glucose regulation and fatty acid oxidation [28]. Adiponectin exerts its actions in the periphery, mainly via two receptors, adiponectin receptor 1 (AdipoR1) and adiponectin receptor 2 (AdipoR2). Visfatin is an enzyme encoded by the NAMPT gene (nicotinamide phosphoribosyltransferase) that was first isolated from human peripheral blood lymphocytes. Outside the cell, visfatin functions as a pro-inflammatory adipokine by increasing the production of inflammatory cytokines by monocytes and leukocytes, and by activating nuclear factor kappa B (NFKB) signaling [29,30]. Resistin, also known as adipose tissue-specific secretory factor (ADSF), is a cysteine-rich peptide hormone derived from adipose tissue encoded by the RETN gene. In primates, pigs, and dogs, resistin is secreted by immune and epithelial cells, whereas it is secreted by adipose tissue in rodents [31,32]. Adipokines are a group of hormones and signaling molecules that are secreted by adipose tissue. These molecules signal through various pathways in the body. For example, some adipokines, such as tumor necrosis factor-alpha (TNF-α) and interleukin-6 (IL-6), can activate the nuclear factor kappa-light-chain-enhancer of activated B cells (NF-κB) pathway, which is involved in inflammation and immune function. Adipokines such as leptin and resistin can activate the JAK/STAT pathway, which is involved in inflammation and immune function. Another adipokine, adiponectin, stimulates AMP-activated protein kinase (AMPK) activity, which plays a key role in the regulation of cellular metabolism and energy homeostasis. Adipokines such as adiponectin, leptin, and resistin can affect insulin signaling, which is involved in glucose and lipid metabolism. Additionally, adipokines such as adiponectin and leptin can activate mitogen-activated protein kinases (MAPKs), which play important roles in cell proliferation, differentiation, and apoptosis. Overall, adipokine signaling through these pathways helps to regulate glucose and lipid metabolism, inflammation, immune function, and other important physiological processes in the body.

Adipokines have been shown to play a functional role in mammalian ovarian physiology [33,34]. Moreover, it has been demonstrated that the ovary serves as a target organ for adipokines. Their presence has been confirmed in various ovarian structures, including granulosa, theca, and luteal cells [12]. Studies have revealed that adipokines influence mechanisms associated with steroid synthesis, and can modify the production of progesterone and prostaglandins, which are key regulators of corpus luteum lifespan [35]. Consequently, adipokines may represent a class of factors that contribute to the regulation of the CL function during the estrous cycle and pregnancy.

However, further research is needed to fully elucidate their functions and their associations with other biochemical pathways, particularly in cattle. To address this knowledge gap, our study aimed to investigate the ovarian expression levels and potential regulatory effects of adipokines at different time points in the corpus luteum during the estrous cycle and pregnancy. Specifically, we focused on the local adipokines mentioned above. Through our research, we hope to contribute to a better understanding of the role of adipokines in ovarian physiology, which could have important implications for the reproductive performance of cattle.

## 2. Materials and Methods

### 2.1. Female Reproductive Tract Examination and Corpus Luteum Collection

The CL from dairy cows (German Fleckvieh) were collected from the local slaughterhouses, first frozen in liquid nitrogen, and then frozen at −80 °C until further processing for RNA isolation techniques [36,37]. After macroscopic observation of ovaries and uterus follicles (size, shape), corpus luteum (size, color, cavities, vacuoles), cervix, and uterus in cycling cows, we measured crown–rump length, evaluated placental characteristics (cotyledons, thickness), and examined fetal weight and organ development in pregnant cows [38,39]. Subsequently, the CL were classified into the following stages of the estrous cycle: Group I (Day 1–2, *n* = 10), Group II (Day 3–4, *n* = 10), Group III (Day 5–7, *n* = 10), Group IV (Day 8–12, *n* = 10), Group V (Day 13–18, *n* = 10), Group VI (Day > 18, *n* = 10). In addition, four groups of pregnancy stages were formed: Group VII (Month 1 and 2 of pregnancy, *n* = 6), Group VIII (Month 3–4, *n* = 6), Group IX (Month 5–7, *n* = 9), Group X (Month > 7, *n* = 7).

### 2.2. RNA Extraction and RT-qPCR

Total RNA was extracted from 50 mg tissue of the dissected corpora lutea using a liquid–liquid extraction protocol (acid guanidinium thiocyanate–phenol–chloroform extraction) with QIAzol^®^ Lysis Reagent (Qiagen, Hilden, Germany). The total RNA pellet was dissolved in 100 µL nuclease-free water. The RNA concentration was determined by measuring the absorbance at 260 nm and a ratio of 260/280 nm using a NanoDrop^®^ ND-1000 Spectrophotometer (Thermo Fisher Scientific, Waltham, MA, USA). All samples showed high RNA purity and a 260/280 ratio of >2. Additionally, RNA integrity was assessed using an Agilent 2100 Bioanalyzer (Agilent Technologies, Santa Clara, CA, USA), which classifies RNA integrity into a score called the RNA Integrity Number (RIN). The automatically calculated RNA Integrity Number (RIN), using the ratio of 28S to 18S rRNA, ranks RNA integrity on a scoring system from 1 to 10, with 1 being the most degraded total RNA profile and 10 being the most intact total RNA extract. In our experiments, we depicted samples in which RIN was always higher than 5.

Primers and oligonucleotide probes (hydrolysis) for housekeeping genes, i.e., ubiquitin, ubiquitin C, cyclophilin A, and target genes, i.e., vaspin, heat shock 70 kDa protein 5 (HSPA5), adiponectin, adipoR1, adipoR2, visfatin, and resistin, were designed using the Primer3 program (version 4.1.0) and the databases from Ensembl (EMBL-EBI) [40]. Moreover, primers were analyzed using NetPrimer (Premier Biosoft) to avoid homologies and template secondary structures. Primer and probe sequence similarities were checked using the Standard Nucleotide BLAST (NCBI) program, and all primers were specific to the target sequences (Table 1). Primers were ordered from Biomers.net (accessed on 2 February 2022) GmbH (Ulm, Germany).

The total extracted RNA was diluted to the desired concentration of 30 ng/µL for mRNA transcript quantification via RT-qPCR. We applied a one-step real-time quantification using hydrolysis probes (Luna^®^ Universal Probe One-PCR) in a 10 µL volume reaction containing 5 µL Luna Universal Probe One-Step Reaction Mix (New England BioLabs Inc., Ipswich, MA, USA), 0.5 µL Luna WarmStart RT Enzyme Mix, 0.4 µL each of forward and reverse primer of the targeted sequence (10 µM), 0.2 µL hydrolysis probe of the corresponding sequence, 3.0 µL of sample RNA, and 0.5 µL of nuclease-free water. The following cycling protocol was applied in a thermal cycler (Rotor-Gene Q, model 5-Plex HRM, Qiagen): one cycle of reverse transcription at 55 °C for 10 min, one cycle of initial denaturation at 95 °C, forty-five cycles of denaturation at 95 °C for 10 s, and extension at 60 °C for 30 s. RT-qPCR products were checked in 2% agarose gel via electrophoresis (VWR International, Darmstadt, Germany) to see if the RT-qPCR product length corresponded with the calculated and expected target sequence lengths. The complete RT-qPCR workflow complied with the MIQE guidelines [41].

### 2.3. Determination of Relative mRNA Expression

Threshold data (Cq values) were obtained via Rotor-Gene Rotor Q Software 2.3.1 (Qiagen, Hilden, Germany) using the comparative quantitation method. The mRNA expression levels were normalized to the geometric mean of three constantly expressed reference genes: cyclophilin A (PPIA), ubiquitin, and ubiquitin C. The geometric mean, when checked in the geNorm and NormFinder algorithms, on GenEX software (version 7)was always the most stable and constantly expressed normalizer (please see the Supplementary Materials (Figures S1–S4). The geNorm algorithm calculates the expression stability of candidate reference genes by comparing their average pairwise variation with that of other reference genes. Genes with the lowest pairwise variation are considered the most stable and are recommended for the normalization of gene expression data. The NormFinder algorithm, on the other hand, calculates a stability value for each candidate reference gene, considering both intragroup and intergroup variations. Genes with lower stability values are considered more stable, and are recommended for the normalization of gene expression data [42,43].

Thus, after the data were normalized with the geometric mean of the housekeeping genes, we compared the normalized difference in expression, using the method known as ∆∆Cq, between two sample groups: the “treatment group” and the “control group” [44]. The relative gene expression (depicted in fold change regulation) was implemented to determine the mRNA expression difference between a treatment group in comparison to a control group [44]. The first group of cycling and pregnant cows was used as the control group, and was set to 1.0. All mRNA expression results are shown as x-fold regulation ± ∆Cq SEM per group.

### 2.4. Statistical Analysis

In this study, we used corpora lutea from cycling cows divided into six groups and pregnant cows into four groups. The cycling cow group consists of the following stages: Group I (Day 1–2, *n* = 10), Group II (Day 3–4, *n* = 10), and Group III (Day 5–7, *n* = 10), Group IV (Day 8–12, *n* = 10), Group V (Day 13–18, *n* = 10), Group VI (Day > 18, *n* = 10). Samples from pregnant cows were divided into the following groups: Group VII (Months 1 and 2 of pregnancy, *n* = 6), Group VIII (Months 3–4, *n* = 6), Group IX (Months 5–7, *n* = 9), and Group X (Months > 7, *n* = 7). All the data are shown as ΔCq means ± SEM (standard error of the mean). Statistical significance was assessed by one way ANOVA followed by the Tukey’s HSD and LSD for multiple comparison analysis. Changes were considered significant if they exhibited a *p*-value < 0.05.

## 3. Results

**Figure 1:**
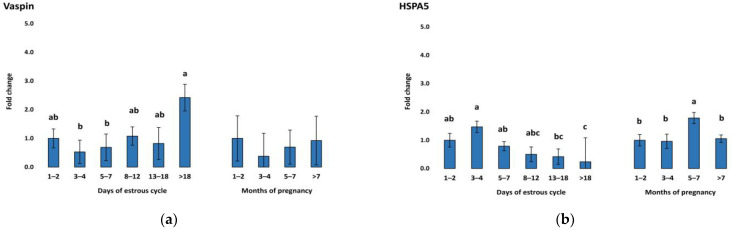
Relative messenger RNA expression of (**a**) vaspin and its receptor (**b**) HSPA5 (heat shock protein 5) and in the CL during the estrous cycle (*n* = 10) and pregnancy (*n* = 6–9). Threshold data were normalized to the geometric mean of the three reference genes (cyclophilin A, ubiquitin, and ubiquitin C). The data are plotted as fold change ± SEM of group ∆Cq. The first group of cycling (D1–2) and pregnant cows (M1–2) were used as control groups. The different significance levels are indicated by different superscripts (*p* < 0.05). SEM, standard error of the mean.

**Figure 2:**
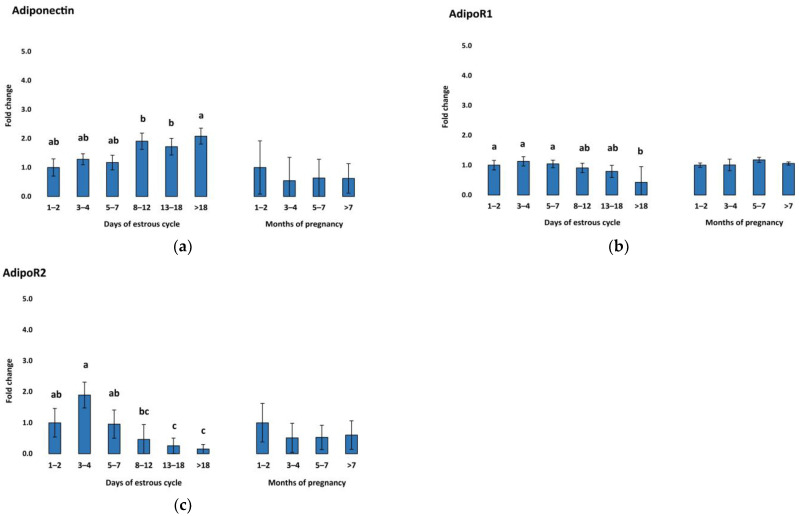
Relative messenger RNA expression of (**a**) adiponectin and its two receptors (**b**) adiponectin receptor 1 and (**c**) adiponectin receptor 2 in the CL during the estrous cycle (*n* = 10) and pregnancy (*n* = 6–9). Threshold data were normalized to the geometric mean of the three reference genes (cyclophilin A, ubiquitin, and ubiquitin C). The data are plotted as fold change ± SEM of group ∆Cq. The first group of cycling (D1–2) and pregnant cows (M1–2) were used as control groups. The different significance levels are indicated by different superscripts (*p* < 0.05). SEM, standard error of the mean.

**Figure 3:**
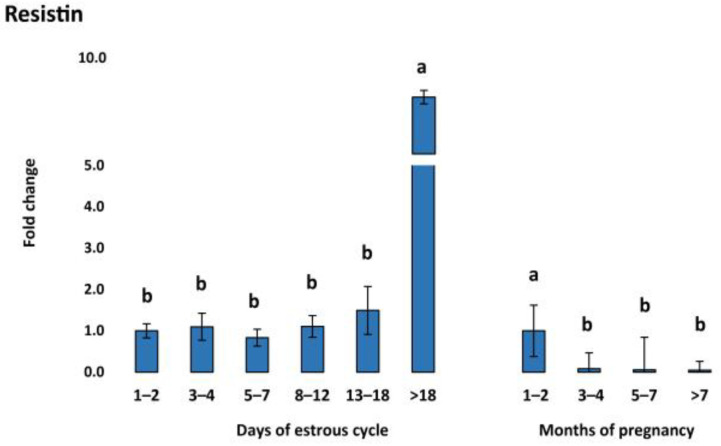
Relative messenger RNA expression of resistin in the CL during the estrous cycle (*n* = 10) and pregnancy (*n* = 6–9). Threshold data were normalized to the geometric mean of the three reference genes (cyclophilin A, ubiquitin, and ubiquitin C). The data are plotted as fold change ± SEM of group ∆Cq. The first group of cycling (D1–2) and pregnant cows (M1–2) were used as control groups. The different significance levels are indicated by different superscripts (*p* < 0.05). SEM, standard error of the mean.

Expression of vaspin mRNA in the CL was downregulated in early luteal stages (D1–2, D3–4, D5–7) followed by a continuous increase, with the highest expression in the regression stage (D > 18) of the estrous cycle (Figure 1a). Meanwhile, vaspin was constantly expressed in the CL during pregnancy without any significant regulation pattern. In contrast, HSPA5 mRNA concentration, the gene responsible for producing the vaspin receptor, was downregulated during the late stages (D13–18, D > 18) of the cycle and upregulated in earlier stages (D1–2 and D3–4) (Figure 1b). Adiponectin showed a similar pattern to vaspin during the estrous cycle, being upregulated in late stages D8–12 and D13–18, with the highest expression in the regression stage (D > 18) (Figure 2a), while being expressed constantly during pregnancy. In addition, AdipoR1 showed slight upregulation in the early luteal stages (D1–2, D3–4, D5–7) and late luteal stage (D13–18) (Figure 2b). AdipoR2 showed a different pattern with high expression in early luteal stages (D1–2, D3–4, and D5–7) (Figure 2c). In our study, resistin was significantly upregulated during the regression stage (D > 18) and in the first months of pregnancy (Figure 3). The mRNA expression of visfatin in our CL groups during the estrous cycle and pregnancy showed no statistically significant changes.

## 4. Discussion

Different studies have shown that various metabolism-related factors and hormones can affect and regulate ovarian function [45,46]. Remembering that adipose tissue, additional to its role in fat storage, acts as an endocrine organ, and its relation to other metabolites, in particular insulin, indicates that a regulatory role in local regulation is very likely [47,48]. There is a strong link between the energy balance and the female reproductive tract, hence, abnormalities in energy homeostasis can lead to different reproductive pathophysiological phenomena. Several studies have shown a link between adipokines and pathologies such as polycystic ovarian syndrome and endometriosis [49,50].

Currently, there are limited data available regarding the expression and function of vaspin in reproductive physiology, particularly in the CL or bovine ovary. As a result, our interpretation and understanding of its role is insufficient. However, studies have shown that vaspin and HSPA5, both in terms of mRNA and protein expression, are predominantly found in porcine ovary structures, such as ovarian follicles, oocytes, and the CL [51]. In these studies, it was found that vaspin may play a critical role in angiogenesis, proliferation, and luteal cell apoptosis in the porcine ovary [52]. Our findings are consistent with these studies, as we observed similar patterns of expression for vaspin and HSPA5 to those in the porcine CL, with higher expression levels in the middle and late stages (Figure 1a), suggesting an involvement in CL steroidogenesis and luteolysis [51]. Vaspin has been shown to bind to the cell surface of a 78 kDa glucose-regulated protein (GRP78), which is also referred to as the heat shock protein family member 5 (HSPA5) or binding immunoglobulin protein. HSPAS mRNA expression in our experiment (Figure 1b) is consistent with some previous studies, indicating that HSPA5 is highly expressed during the early and middle stages of CL, followed by a decrease in the regression stage [53]. This suggests that HSPA5, as one of the most well-studied endoplasmic reticulum (ER) chaperone proteins, may function as an ER chaperone to establish and maintain the CL’s resistance to caspase activation during the early and middle stages of CL. In rat CL regression induced by prostaglandin F2 alpha, the expression level of HSPA5 increases from the early stage, reaching its highest expression level in the regression stage [54].

Research has shown that adiponectin plays a crucial role in oocyte maturation, granulosa cell proliferation, and steroid secretion in the ovaries [23]. Additionally, adiponectin has been suggested to influence angiogenic factors in the development of porcine CL [55]. Previous studies have reported higher adiponectin levels during the follicular stage, with particularly high levels in cumulus–oocyte complexes and theca cells of dominant follicles [56]. Our findings are consistent with those of Tabandeh et al. [57]. Both studies show high adiponectin expression during the bovine CL regression stage and a subsequent decrease during the onset of CL growth (Figure 2a). Moreover, there is evidence of adiponectin in the late stages of folliculogenesis and development of a functional placenta [33]. Adiponectin receptor 1 demonstrated a similar pattern (Figure 2b), with higher expression during the early and middle luteal lifespan [57]. However, in contrast to Tabandeh et al.’s findings [57], our study observed higher expression levels of adiponectin receptor 2 in the early stages of the CL lifespan, rather than in the regression stage (Figure 2c). While limited, these findings suggest a possible connection between adiponectin and the inhibition of progesterone production. This is significant because progesterone is known to be highly produced during the middle stages of CL. Adiponectin have also been shown to be highly expressed in porcine ovarian granulosa cells, where it induces the expression of genes and proteins in a pattern that resembles periovulatory remodeling of the ovarian follicle. Specifically, it induces the expression of cyclooxygenase-2 (COX-2) and prostaglandin E (PGE) [58].

Numerous studies have demonstrated a strong correlation between resistin and female reproductive function [59]. A study conducted on bovine and rat ovaries has demonstrated the expression of resistin. This study revealed that resistin has an inhibitory effect on granulosa cell steroidogenesis in bovine ovaries, but has a stimulatory effect on P4 secretion in rats [60]. In a recent study, it was observed that resistin decreased progesterone (P4) levels, increased estradiol (E2) levels, and regulated various pathways, such as those of steroidogenic acute regulatory protein (STAR), cholesterol side-chain cleavage enzyme, 3β-hydroxysteroid dehydrogenase, estrogen synthetase, through the activation of protein kinase A and mitogen-activated protein kinase 1 [61]. This study provides further evidence for the complex effects of resistin on reproductive function. Specifically, resistin was found to stimulate the expression of luteinizing hormone/choriogonadotropin receptor (LHCGR) and estrogen receptor beta, while having an inhibitory effect on the progesterone receptor (PGR). These findings suggest a potential inhibitory effect on progesterone production. In addition, an in vitro cell culture study demonstrated that resistin inhibits the secretion of estradiol and progesterone from granulosa cells as well as the stimulatory effect of follicle-stimulating hormone on E2 and P4 [62]. Resistin showed inhibitory action on steroidogenesis in theca and granulose cells collected from the ovarian follicles of cycling cattle [63]. Our findings also suggest an increase in resistin in the regression stage of CL and during the first months of pregnancy (Figure 3). Resistin has been identified in human placental tissue, predominantly in trophoblastic cells and the amniotic membrane [64]. During pregnancy, elevated levels of resistin and adipokines are generally linked to an increase in insulin resistance [65]. However, very little is known about the precise role and mechanisms of resistin in ruminants. No resistin-specific receptor is known yet.

In cattle, visfatin has been shown to play a role in the activation of IGF1R and MAPK3/1, resulting in a greater abundance of steroidogenic acute regulatory protein (StAR) and HSD3B activity [66]. A recent study on mice reported an increase in visfatin expression in the CL [67]. Another study showed that recombinant human visfatin, when administered at a concentration of 10 ng/mL, significantly increased progesterone and estradiol secretion induced by IGF-1 in primary human granulosa cells and human ovarian granulosa-like tumor cell line (KGN) cells. However, a specific receptor for visfatin has not yet been identified. Visfatin expression has also been demonstrated in hen granulosa cells, and a study reported that in vitro administration of recombinant human NAMPT inhibited both basal and IGF1-induced progesterone secretion by granulosa cells [68]. In our study, we found that visfatin expression remained constant throughout the estrous cycle and pregnancy without significant changes among the groups.

However, it is important to note that the detection of mature proteins and histological localization of the studied adipokines and their receptors in bovine species is impossible, due to the lack of commercial and stabilized antibodies. The development of specific and reliable antibodies is crucial to gain a better understanding of adipokines at different molecular levels and facilitate their expression strength and exact tissue localization. This will help us speculate about the potential of adipokines in reproductive function and provide valuable insight into their underlying mechanisms. Hence, further research is needed to identify and develop stable antibodies that can accurately measure the expression and localization of adipokines and their receptors in bovine tissues.

Through our investigation, we provide data that contribute towards advancing our understanding of the complex mechanisms that govern dairy cattle reproduction. Our findings give valuable insight into the local regulation of adipokines within the corpus luteum of dairy cattle, offering a deeper understanding of the intricate interplay between adipokines and reproductive physiology. By delving into the local regulation of adipokines within the corpus luteum, we contribute to the existing knowledge base, and highlight the importance of continued research efforts in this domain and the exploration of additional factors that may influence the interplay between adipokines and reproductive processes in dairy cattle.

## 5. Conclusions

Our findings demonstrate that adipokines are present in the CL during all cyclic and pregnancy stages, suggesting their important role in regulating CL function. Moreover, the up- or downregulation of adipokine mRNA levels varied among different stages of the estrous cycle and pregnancy, indicating their potential regulatory effects. The specific expression of adipokines and their receptors in the CL suggests their participation in the local mechanisms that regulate CL activity. In conclusion, our present study suggests that all investigated adipokines are expressed in the bovine CL mRNA during the estrous cycle and pregnancy, and are regulated independently and differently. However, further investigations are required to elucidate the detailed regulation and mechanisms involved in different local actions of adipokines in CL tissue during the estrous cycle and pregnancy. Understanding the role of adipokines in CL function may lead to the development of targeted therapies that can improve fertility and reproductive function in both humans and animals.

## Figures and Tables

**Table 1:** Primer sequence, probe sequences, RT-qPCR product length, and Ensembl transcript ID of amplified genes (vaspin, heat shock protein 5 (HSPA5), adiponectin, adiponectin receptor 1 (AdipoR1), adiponectin receptor 2 (AdipoR2), and resistin) and reference genes (ubiquitin (UBA), ubiquitin C (UBC), and cyclophilin A (PPIA)).

Target	Sequence of Nucleotides *	Amplicon Size (bp)	Ensembl Transcript
PPIA	For-5′-CTGAGCACTGGAGAGAAAGGA-3′	116	ENSBTAT00000015924.5
	Rev-5′-GACTTGCCACCAGTACCATT-3′		
	Hyb-5′-TCCGGGATTTATGTGCCAGGGTGGT-3′		
UBQ	For-5′-GGCTGATCTTCGCTGGCA-3′	124	ENSBTAT00000010176.3
	Rev-5′-CGGAGGGAAGGCTCGATG-3′		
	Hyb-5′-TGGAGGATGGCCGCACTCTGTCAGA-3′		
UBC	For-5′-GACCGGGAGTTCAGTCTTCG-3′	133	ENSBTAT00000046011.4
	Rev-5′-TTCTCGATGGTGTCACTGGG-3′		
	Hyb-5′-TGTGTTCGCTGCTGACACCACCACT-3′		
Vaspin	For-5′-TACCAGAGCAACTTCACGGC-3′	146	ENSBTAT00000045204.4
	Rev-5′-GTCAACCTGGGCACAAACAC-3′		
	Hyb-5′-TGAAGCAAGTGGAGCAAGCCCTGGG-3′		
HSPA5	For-5′-TTTCTGCCATGGTTCTCACT-3′	125	ENSBTAT00000057533.3
	Rev-5′-ATCTTTGGTTGCCTGGCGTT-3′		
	Hyb-5′-AGGAAACTGCTGAGGCTTATTTGGGA-3′		
Adiponectin	For-5′-GTGAGAAGGGTGAGAAAGGAGA-3′	136	ENSBTAT00000077795.1
	Rev-5′-GCACTTTCTCCAGGTTCTCCC-3′		
	Hyb-5′-GAGGCTTTCCAGGAACCCCAGGCAG-3′		
AdipoR1	For-5′-CCACACTGTCTACTGTCATTCA-3′	150	ENSBTAT00000040283.5
	Rev-5′-GAGAGGTAGATGAGCCGAGG-3′		
	Hyb-5′-TGCTGATCATGGGGAGCTTCGTGCC-3′		
AdipoR2	For-5′-GGCGTCTGTCCTTTCTTCCT-3′	110	ENSBTAT00000011923.6
	Rev-5′-CTTGCAGGAGAGGGGACATG-3′		
	Hyb-5′-GGAGCGTGAGTGCGATGATGGAGCA-3′		
Resistin	For-5′-CAGTCGCTGTGCCCCATAG-3′	91	ENSBTAT00000006189.3
	Rev-5′-GGCCAATGATCCTTACTGCC-3′		
	Hyb-5′-TGAGAAGATCCAGGAGGTCACCACC-3′		
Visfatin	For-5′-TCGAAGGGCTACAAGTTGCT-3′	143	ENSBTAT00000020608.4
	Rev-5′-GCTCCACCAGAACCAAAGGA-3′		
	Hyb-5′-ACAAGAGATTGTGGAAGGCATGAAGCA-3′		

* For, forward primer; Rev, reverse primer; Hyb, hybridization probe.

## Data Availability

The detailed results can be found in the Supplementary Materials. Further data supporting this study’s findings are available from the corresponding author upon reasonable request.

## Supplementary Materials

The following supporting information can be downloaded at https://www.mdpi.com/article/10.3390/ani13111782/s1. Figure S1: Classification of target genes on estrous cycle groups based on geNorm algorithm. Figure S2: Classification of target genes on pregnancy groups based on geNorm algorithm. Figure S3: Classification of target genes on estrous cycle groups based on NormFinder algorithm. Figure S4: Classification of target genes on pregnancy groups based on NormFinder algorithm. Table S1. Statistical significance between estrous cycle sampling groups was analyzed through multiple comparison tests (Tukey´s HSD, LSD). Table S2. Statistical significance between pregnancy sampling groups was analyzed through multiple comparison tests (Tukey´s HSD, LSD).
