# Supplementary material for: Expression of Locally Produced Adipokines and Their Receptors during Different Physiological and Reproductive Stages in the Bovine Corpus Luteum
Source: Animals (Basel). 2023 May 27;13(11):1782. doi: 10.3390/ani13111782 (PMC10251875; doi:10.3390/ani13111782)
Supplement: Supplementary file 1 [file animals-13-01782-s001.zip › animals-2374325-supplementary.pdf]

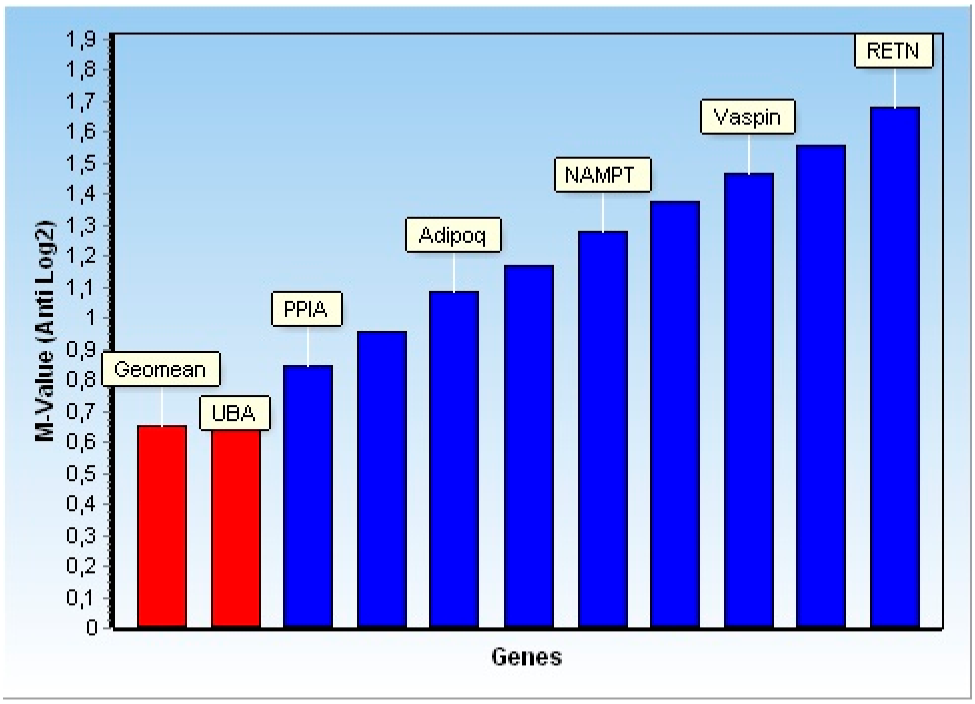


Figure S1: Classification of target genes on estrous cycle groups based on geNorm algorithm.

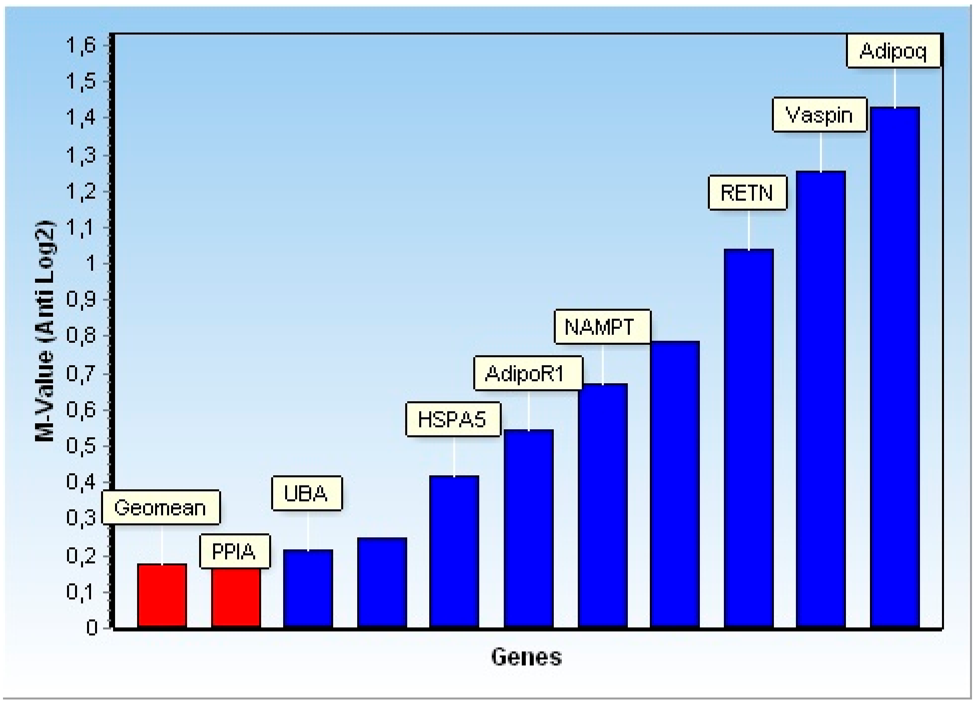


Figure S2: Classification of target genes on pregnancy groups based on geNorm algorithm.

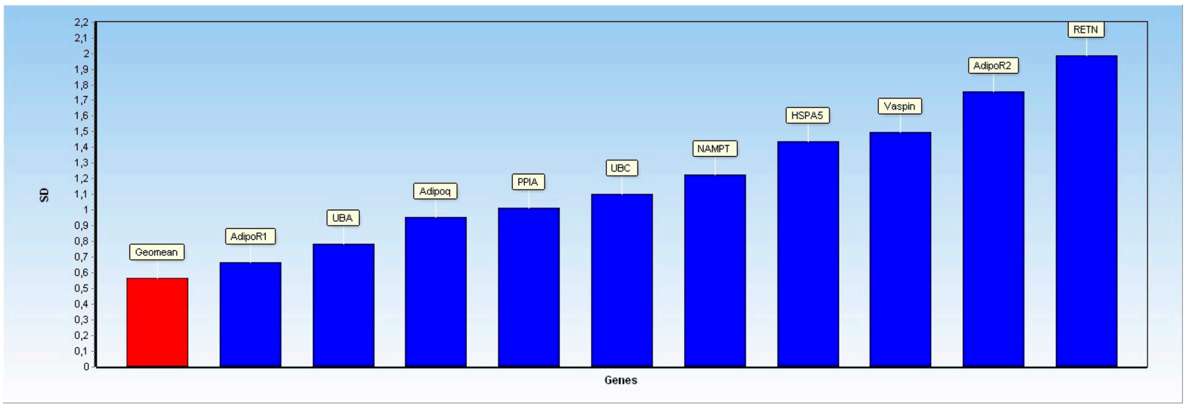


Figure S3: Classification of target genes on estrous cycle groups based on NormFinder algorithm.

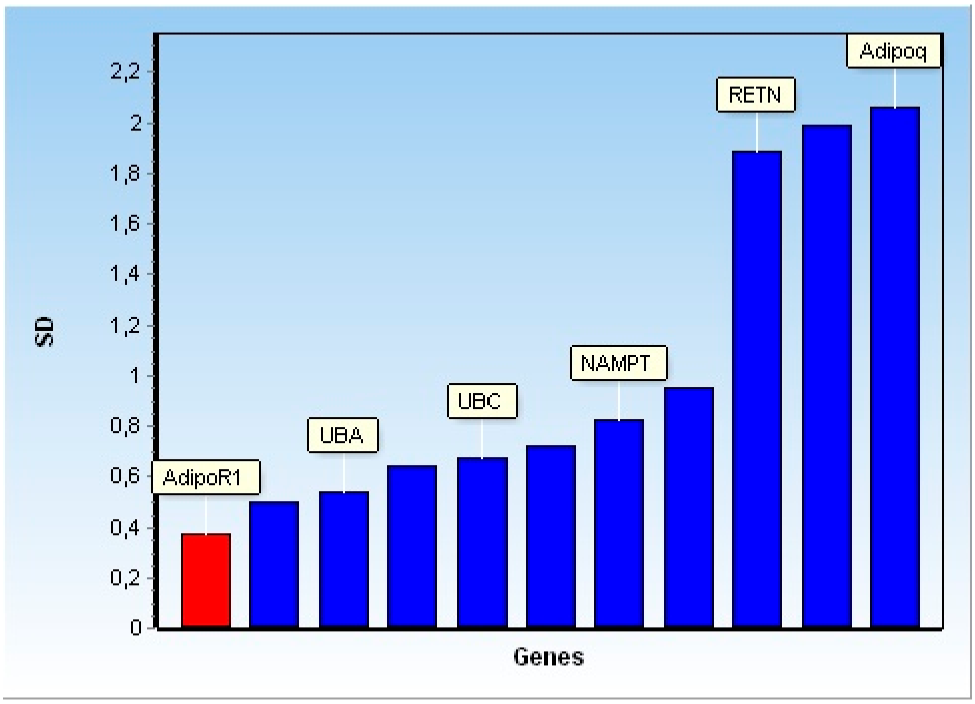


Figure S4: Classification of target genes on pregnancy groups based on NormFinder algorithm.
