# Supplementary material for: Expression of Locally Produced Adipokines and Their Receptors during Different Physiological and Reproductive Stages in the Bovine Corpus Luteum
Source: Animals (Basel). 2023 May 27;13(11):1782. doi: 10.3390/ani13111782 (PMC10251875; doi:10.3390/ani13111782)
Supplement: Supplementary file 1 [file animals-13-01782-s001.zip › Table S1. Multiple comparisons -Estrous cycle.pdf]

Multiple Comparisons between estrous cycle sampling groups

Dependent Variable		(I) #Treatment	(J) #Treatment	Mean Difference (I-J)	Std. Error	Sig.
Vaspin	Tukey HSD	1	2	-.6105404406	.56273381880	,885
			3	-.2368658003	.56273381880	,998
			4	.41537095723	.56273381880	,976
			5	.31522088014	.56273381880	,993
			6	1.5816098491	.56273381880	,071
		2	1	.61054044061	.56273381880	,885
			3	.37367464036	.56273381880	,985
			4	1.0259113978	.56273381880	,460
			5	.92576132075	.56273381880	,573
			6	2.192150290*	.56273381880	,004
		3	1	.23686580025	.56273381880	,998
			2	-.3736746404	.56273381880	,985
			4	.65223675748	.56273381880	,854
			5	.55208668039	.56273381880	,922
			6	1.818475649*	.56273381880	,024
		4	1	-.4153709572	.56273381880	,976
			2	-1.025911398	.56273381880	,460
			3	-.6522367575	.56273381880	,854
			5	-.1001500771	.56273381880	1,000
			6	1.1662388919	.56273381880	,317
		5	1	-.3152208801	.56273381880	,993
			2	-.9257613207	.56273381880	,573
			3	-.5520866804	.56273381880	,922
			4	.10015007709	.56273381880	1,000
			6	1.2663889690	.56273381880	,233
		6	1	-1.581609849	.56273381880	,071
			2	-2.192150290*	.56273381880	,004
			3	-1.818475649*	.56273381880	,024
			4	-1.166238892	.56273381880	,317
			5	-1.266388969	.56273381880	,233
	LSD	1	2	-.6105404406	.56273381880	,283
			3	-.2368658003	.56273381880	,675
			4	.41537095723	.56273381880	,464
			5	.31522088014	.56273381880	,578
			6	1.581609849*	.56273381880	,007
		2	1	.61054044061	.56273381880	,283
			3	.37367464036	.56273381880	,509
			4	1.0259113978	.56273381880	,074
			5	.92576132075	.56273381880	,106
			6	2.192150290*	.56273381880	<,001

Multiple Comparisons between estrous cycle sampling groups

Dependent Variable		(I) #Treatment	(J) #Treatment	95% Confidence Interval Lower Bound	95% Confidence Interval Upper Bound
Vaspin	Tukey HSD	1	2	-2.273126067	1.0520451861
			3	-1.899451427	1.4257198264
			4	-1.247214669	2.0779565839
			5	-1.347364747	1.9778065068
			6	-.0809757776	3.2441954758
		2	1	-1.052045186	2.2731260673
			3	-1.288910986	2.0362602671
			4	-.6366742289	2.6884970245
			5	-.7368243060	2.5883469474
			6	.52956466303	3.8547359164
		3	1	-1.425719826	1.8994514269
			2	-2.036260267	1.2889109863
			4	-1.010348869	2.3148223842
			5	-1.110498946	2.2146723071
			6	.15589002268	3.4810612761
		4	1	-2.077956584	1.2472146695
			2	-2.688497025	.63667422886
			3	-2.314822384	1.0103488692
			5	-1.762735704	1.5624355496
			6	-.4963467348	2.8288245186
		5	1	-1.977806507	1.3473647466
			2	-2.588346947	.73682430595
			3	-2.214672307	1.1104989463
			4	-1.562435550	1.7627357038
			6	-.3961966577	2.9289745957
		6	1	-3.244195476	.08097577758
			2	-3.854735916	-.5295646630
			3	-3.481061276	-.1558900227
			4	-2.828824519	.49634673480
			5	-2.928974596	.39619665771
	LSD	1	2	-1.738753819	.51767293747
			3	-1.365079178	.89134757783
			4	-.7128424209	1.5435843353
			5	-.8129924979	1.4434342582
			6	.45339647104	2.7098232272
		2	1	-.5176729375	1.7387538187
			3	-.7545387377	1.5018880184
			4	-.1023019802	2.1541247759
			5	-.2024520573	2.0539746988
			6	1.0639369116	3.3203636678

Multiple Comparisons between estrous cycle sampling groups

Dependent Variable		(I) #Treatment	(J) #Treatment	Mean Difference (I-J)	Std. Error	Sig.
		3	1	.23686580025	.56273381880	,675
			2	-.3736746404	.56273381880	,509
			4	.65223675748	.56273381880	,252
			5	.55208668039	.56273381880	,331
			6	1.818475649*	.56273381880	,002
		4	1	-.4153709572	.56273381880	,464
			2	-1.025911398	.56273381880	,074
			3	-.6522367575	.56273381880	,252
			5	-.1001500771	.56273381880	,859
			6	1.166238892*	.56273381880	,043
		5	1	-.3152208801	.56273381880	,578
			2	-.9257613207	.56273381880	,106
			3	-.5520866804	.56273381880	,331
			4	.10015007709	.56273381880	,859
			6	1.266388969*	.56273381880	,029
		6	1	-1.581609849*	.56273381880	,007
			2	-2.192150290*	.56273381880	<,001
			3	-1.818475649*	.56273381880	,002
			4	-1.166238892*	.56273381880	,043
			5	-1.266388969*	.56273381880	,029
HSPA5	Tukey HSD	1	2	.55845955939	.56139378430	,918
			3	-.3408658003	.56139378430	,990
			4	-.9946290428	.56139378430	,492
			5	-1.247120146	.56139378430	,245
			6	-2.077390151*	.56139378430	,006
		2	1	-.5584595594	.56139378430	,918
			3	-.8993253596	.56139378430	,601
			4	-1.553088602	.56139378430	,079
			5	-1.805579705*	.56139378430	,025
			6	-2.635849710*	.56139378430	<,001
		3	1	.34086580025	.56139378430	,990
			2	.89932535964	.56139378430	,601
			4	-.6537632425	.56139378430	,851
			5	-.9062543453	.56139378430	,593
			6	-1.736524351*	.56139378430	,035
		4	1	.99462904277	.56139378430	,492
			2	1.5530886022	.56139378430	,079
			3	.65376324252	.56139378430	,851
			5	-.2524911028	.56139378430	,998
			6	-1.082761108	.56139378430	,396

Multiple Comparisons between estrous cycle sampling groups

Dependent Variable		(I) #Treatment	(J) #Treatment	95% Confidence Interval Lower Bound	Upper Bound
		3	1	-.8913475778	1.3650791783
			2	-1.501888018	.75453873773
			4	-.4759766206	1.7804501356
			5	-.5761266977	1.6803000585
			6	.69026227129	2.9466890275
		4	1	-1.543584335	.71284242085
			2	-2.154124776	.10230198025
			3	-1.780450136	.47597662060
			5	-1.228363455	1.0280633010
			6	.03802551381	2.2944522700
		5	1	-1.443434258	.81299249794
			2	-2.053974699	.20245205734
			3	-1.680300058	.57612669769
			4	-1.028063301	1.2283634552
			6	.13817559090	2.3946023471
		6	1	-2.709823227	-.4533964710
			2	-3.320363668	-1.063936912
			3	-2.946689027	-.6902622713
			4	-2.294452270	-.0380255138
			5	-2.394602347	-.1381755909
HSPA5	Tukey HSD	1	2	-1.100166963	2.2170860814
			3	-1.999492322	1.3177607218
			4	-2.653255565	.66399747924
			5	-2.905746668	.41150637649
			6	-3.736016673	-.4187636289
		2	1	-2.217086081	1.1001669626
			3	-2.557951882	.75930116237
			4	-3.211715124	.10553791985
			5	-3.464206227	-.1469531829
			6	-4.294476232	-.9772231883
		3	1	-1.317760722	1.9994923223
			2	-.7593011624	2.5579518817
			4	-2.312389765	1.0048632795
			5	-2.564880867	.75237217674
			6	-3.395150873	-.0778978286
		4	1	-.6639974792	2.6532555648
			2	-.1055379198	3.2117151242
			3	-1.004863279	2.3123897645
			5	-1.911117625	1.4061354193
			6	-2.741387630	.57586541391

Multiple Comparisons between estrous cycle sampling groups

Dependent Variable		(I) #Treatment	(J) #Treatment	Mean Difference (I-J)	Std. Error	Sig.
		5	1	1.2471201455	.56139378430	,245
			2	1.805579705*	.56139378430	,025
			3	.90625434527	.56139378430	,593
			4	.25249110275	.56139378430	,998
			6	-.8302700054	.56139378430	,679
		6	1	2.077390151*	.56139378430	,006
			2	2.635849710*	.56139378430	<,001
			3	1.736524351*	.56139378430	,035
			4	1.0827611081	.56139378430	,396
			5	.83027000535	.56139378430	,679
	LSD	1	2	.55845955939	.56139378430	,324
			3	-.3408658003	.56139378430	,546
			4	-.9946290428	.56139378430	,082
			5	-1.247120146*	.56139378430	,031
			6	-2.077390151*	.56139378430	<,001
		2	1	-.5584595594	.56139378430	,324
			3	-.8993253596	.56139378430	,115
			4	-1.553088602*	.56139378430	,008
			5	-1.805579705*	.56139378430	,002
			6	-2.635849710*	.56139378430	<,001
		3	1	.34086580025	.56139378430	,546
			2	.89932535964	.56139378430	,115
			4	-.6537632425	.56139378430	,249
			5	-.9062543453	.56139378430	,112
			6	-1.736524351*	.56139378430	,003
		4	1	.99462904277	.56139378430	,082
			2	1.553088602*	.56139378430	,008
			3	.65376324252	.56139378430	,249
			5	-.2524911028	.56139378430	,655
			6	-1.082761108	.56139378430	,059
		5	1	1.247120146*	.56139378430	,031
			2	1.805579705*	.56139378430	,002
			3	.90625434527	.56139378430	,112
			4	.25249110275	.56139378430	,655
			6	-.8302700054	.56139378430	,145
		6	1	2.077390151*	.56139378430	<,001
			2	2.635849710*	.56139378430	<,001
			3	1.736524351*	.56139378430	,003
			4	1.0827611081	.56139378430	,059
			5	.83027000535	.56139378430	,145

Multiple Comparisons between estrous cycle sampling groups

Dependent Variable		(I) #Treatment	(J) #Treatment	95% Confidence Interval Lower Bound	Upper Bound
		5	1	-.4115063765	2.9057466675
			2	.14695318291	3.4642062269
			3	-.7523721767	2.5648808673
			4	-1.406135419	1.9111176248
			6	-2.488896527	.82835651666
		6	1	.41876362887	3.7360166729
			2	.97722318826	4.2944762323
			3	.07789782861	3.3951508726
			4	-.5758654139	2.7413876301
			5	-.8283565167	2.4888965274
	LSD	1	2	-.5670672113	1.6839863301
			3	-1.466392571	.78466097042
			4	-2.120155813	.13089772790
			5	-2.372646916	-.1215933749
			6	-3.202916922	-.9518633802
		2	1	-1.683986330	.56706721128
			3	-2.024852130	.22620141102
			4	-2.678615373	-.4275618315
			5	-2.931106476	-.6800529342
			6	-3.761376481	-1.510322940
		3	1	-.7846609704	1.4663925709
			2	-.2262014110	2.0248521303
			4	-1.779290013	.47176352815
			5	-2.031781116	.21927242540
			6	-2.862051121	-.6109975800
		4	1	-.1308977279	2.1201558134
			2	.42756183149	2.6786153728
			3	-.4717635282	1.7792900132
			5	-1.378017873	.87303566791
			6	-2.208287879	.04276566256
		5	1	.12159337486	2.3726469162
			2	.68005293425	2.9311064756
			3	-.2192724254	2.0317811159
			4	-.8730356679	1.3780178734
			6	-1.955796776	.29525676532
		6	1	.95186338021	3.2029169215
			2	1.5103229396	3.7613764809
			3	.61099757996	2.8620511213
			4	-.0427656626	2.2082878788
			5	-.2952567653	1.9557967760

Multiple Comparisons between estrous cycle sampling groups

Dependent Variable		(I) #Treatment	(J) #Treatment	Mean Difference (I-J)	Std. Error	Sig.
Adipoq	Tukey HSD	1	2	.51045955939	.33772463897	,659
			3	.37913419975	.33772463897	,870
			4	1.080370957*	.33772463897	,027
			5	1.101220880*	.33772463897	,022
			6	1.207609849*	.33772463897	,009
		2	1	-.5104595594	.33772463897	,659
			3	-.1313253596	.33772463897	,999
			4	.56991139784	.33772463897	,546
			5	.59076132075	.33772463897	,506
			6	.69715028973	.33772463897	,321
		3	1	-.3791341997	.33772463897	,870
			2	.13132535964	.33772463897	,999
			4	.70123675748	.33772463897	,315
			5	.72208668039	.33772463897	,284
			6	.82847564937	.33772463897	,157
		4	1	-1.080370957*	.33772463897	,027
			2	-.5699113978	.33772463897	,546
			3	-.7012367575	.33772463897	,315
			5	.02084992291	.33772463897	1,000
			6	.12723889189	.33772463897	,999
		5	1	-1.101220880*	.33772463897	,022
			2	-.5907613207	.33772463897	,506
			3	-.7220866804	.33772463897	,284
			4	-.0208499229	.33772463897	1,000
			6	.10638896898	.33772463897	1,000
		6	1	-1.207609849*	.33772463897	,009
			2	-.6971502897	.33772463897	,321
			3	-.8284756494	.33772463897	,157
			4	-.1272388919	.33772463897	,999
			5	-.1063889690	.33772463897	1,000
	LSD	1	2	.51045955939	.33772463897	,136
			3	.37913419975	.33772463897	,267
			4	1.080370957*	.33772463897	,002
			5	1.101220880*	.33772463897	,002
			6	1.207609849*	.33772463897	<,001
		2	1	-.5104595594	.33772463897	,136
			3	-.1313253596	.33772463897	,699
			4	.56991139784	.33772463897	,097
			5	.59076132075	.33772463897	,086
			6	.6971502897*	.33772463897	,044

Multiple Comparisons between estrous cycle sampling groups

Dependent Variable		(I) #Treatment	(J) #Treatment	95% Confidence Interval Lower Bound	Upper Bound
Adipoq	Tukey HSD	1	2	-.4873410202	1.5082601389
			3	-.6186663798	1.3769347793
			4	.08257037768	2.0781715368
			5	.10342030059	2.0990214597
			6	.20980926958	2.2054104287
		2	1	-1.508260139	.48734102015
			3	-1.129125939	.86647521990
			4	-.4278891817	1.5677119774
			5	-.4070392588	1.5885619003
			6	-.3006502898	1.6949508693
		3	1	-1.376934779	.61866637980
			2	-.8664752199	1.1291259392
			4	-.2965638221	1.6990373370
			5	-.2757138992	1.7198872599
			6	-.1693249302	1.8262762289
		4	1	-2.078171537	-.0825703777
			2	-1.567711977	.42788918171
			3	-1.699037337	.29656382206
			5	-.9769506566	1.0186505025
			6	-.8705616877	1.1250394714
		5	1	-2.099021460	-.1034203006
			2	-1.588561900	.40703925880
			3	-1.719887260	.27571389916
			4	-1.018650502	.97695065664
			6	-.8914116106	1.1041895485
		6	1	-2.205410429	-.2098092696
			2	-1.694950869	.30065028982
			3	-1.826276229	.16932493017
			4	-1.125039471	.87056168765
			5	-1.104189549	.89141161056
	LSD	1	2	-.1666375744	1.1875566932
			3	-.2979629340	1.0562313335
			4	.40327382345	1.7574680910
			5	.42412374636	1.7783180139
			6	.53051271534	1.8847069829
		2	1	-1.187556693	.16663757439
			3	-.8084224934	.54577177414
			4	-.1071857359	1.2470085316
			5	-.0863358130	1.2678584545
			6	.02005315595	1.3742474235

Multiple Comparisons between estrous cycle sampling groups

Dependent Variable		(I) #Treatment	(J) #Treatment	Mean Difference (I-J)	Std. Error	Sig.
		3	1	-.3791341997	.33772463897	,267
			2	.13132535964	.33772463897	,699
			4	.7012367575*	.33772463897	,043
			5	.7220866804*	.33772463897	,037
			6	.8284756494*	.33772463897	,017
		4	1	-1.080370957*	.33772463897	,002
			2	-.5699113978	.33772463897	,097
			3	-.7012367575*	.33772463897	,043
			5	.02084992291	.33772463897	,951
			6	.12723889189	.33772463897	,708
		5	1	-1.101220880*	.33772463897	,002
			2	-.5907613207	.33772463897	,086
			3	-.7220866804*	.33772463897	,037
			4	-.0208499229	.33772463897	,951
			6	.10638896898	.33772463897	,754
		6	1	-1.207609849*	.33772463897	<,001
			2	-.6971502897*	.33772463897	,044
			3	-.8284756494*	.33772463897	,017
			4	-.1272388919	.33772463897	,708
			5	-.1063889690	.33772463897	,754
AdipoR1	Tukey HSD	1	2	.17545955939	.36860889514	,997
			3	.05913419975	.36860889514	1,000
			4	-.1396290428	.36860889514	,999
			5	-.3371201455	.36860889514	,941
			6	-1.227390151*	.36860889514	,019
		2	1	-.1754595594	.36860889514	,997
			3	-.1163253596	.36860889514	1,000
			4	-.3150886022	.36860889514	,955
			5	-.5125797049	.36860889514	,732
			6	-1.402849710*	.36860889514	,005
		3	1	-.0591341997	.36860889514	1,000
			2	.11632535964	.36860889514	1,000
			4	-.1987632425	.36860889514	,994
			5	-.3962543453	.36860889514	,889
			6	-1.286524351*	.36860889514	,012
		4	1	.13962904277	.36860889514	,999
			2	.31508860216	.36860889514	,955
			3	.19876324252	.36860889514	,994
			5	-.1974911028	.36860889514	,994
			6	-1.087761108	.36860889514	,050

Multiple Comparisons between estrous cycle sampling groups

Dependent Variable		(I) #Treatment	(J) #Treatment	95% Confidence Interval Lower Bound	95% Confidence Interval Upper Bound
		3	1	-1.056231334	.29796293403
			2	-.5457717741	.80842249343
			4	.02413962370	1.3783338913
			5	.04498954661	1.3991838142
			6	.15137851559	1.5055727832
		4	1	-1.757468091	-.4032738234
			2	-1.247008532	.10718573594
			3	-1.378333891	-.0241396237
			5	-.6562472109	.69794705669
			6	-.5498582419	.80433602567
		5	1	-1.778318014	-.4241237464
			2	-1.267858455	.08633581304
			3	-1.399183814	-.0449895466
			4	-.6979470567	.65624721087
			6	-.5707081648	.78348610276
		6	1	-1.884706983	-.5305127153
			2	-1.374247424	-.0200531559
			3	-1.505572783	-.1513785156
			4	-.8043360257	.54985824189
			5	-.7834861028	.57070816480
AdipoR1	Tukey HSD	1	2	-.9135879271	1.2645070459
			3	-1.029913287	1.1481816862
			4	-1.228676529	.94941844371
			5	-1.426167632	.75192734096
			6	-2.316437637	-.1383426644
		2	1	-1.264507046	.91358792709
			3	-1.205372846	.97272212684
			4	-1.404136089	.77395888432
			5	-1.601627191	.57646778157
			6	-2.491897197	-.3138022238
		3	1	-1.148181686	1.0299132867
			2	-.9727221268	1.2053728461
			4	-1.287810729	.89028424397
			5	-1.485301832	.69279314121
			6	-2.375571837	-.1974768641
		4	1	-.9494184437	1.2286765293
			2	-.7739588843	1.4041360886
			3	-.8902842440	1.2878107290
			5	-1.286538589	.89155638373
			6	-2.176808595	.00128637838

Multiple Comparisons between estrous cycle sampling groups

Dependent Variable		(I) #Treatment	(J) #Treatment	Mean Difference (I-J)	Std. Error	Sig.
		5	1	.33712014553	.36860889514	,941
			2	.51257970492	.36860889514	,732
			3	.39625434527	.36860889514	,889
			4	.19749110275	.36860889514	,994
			6	-.8902700054	.36860889514	,169
		6	1	1.227390151*	.36860889514	,019
			2	1.402849710*	.36860889514	,005
			3	1.286524351*	.36860889514	,012
			4	1.0877611081	.36860889514	,050
			5	.89027000535	.36860889514	,169
	LSD	1	2	.17545955939	.36860889514	,636
			3	.05913419975	.36860889514	,873
			4	-.1396290428	.36860889514	,706
			5	-.3371201455	.36860889514	,364
			6	-1.227390151*	.36860889514	,002
		2	1	-.1754595594	.36860889514	,636
			3	-.1163253596	.36860889514	,754
			4	-.3150886022	.36860889514	,396
			5	-.5125797049	.36860889514	,170
			6	-1.402849710*	.36860889514	<,001
		3	1	-.0591341997	.36860889514	,873
			2	.11632535964	.36860889514	,754
			4	-.1987632425	.36860889514	,592
			5	-.3962543453	.36860889514	,287
			6	-1.286524351*	.36860889514	<,001
		4	1	.13962904277	.36860889514	,706
			2	.31508860216	.36860889514	,396
			3	.19876324252	.36860889514	,592
			5	-.1974911028	.36860889514	,594
			6	-1.087761108*	.36860889514	,005
		5	1	.33712014553	.36860889514	,364
			2	.51257970492	.36860889514	,170
			3	.39625434527	.36860889514	,287
			4	.19749110275	.36860889514	,594
			6	-.8902700054*	.36860889514	,019
		6	1	1.227390151*	.36860889514	,002
			2	1.402849710*	.36860889514	<,001
			3	1.286524351*	.36860889514	<,001
			4	1.087761108*	.36860889514	,005
			5	.8902700054*	.36860889514	,019

Multiple Comparisons between estrous cycle sampling groups

Dependent Variable	(I) #Treatment	(J) #Treatment	95% Confidence Interval Lower Bound	95% Confidence Interval Upper Bound
	5	1	-.7519273410	1.4261676320
		2	-.5764677816	1.6016271914
		3	-.6927931412	1.4853018318
		4	-.8915563837	1.2865385892
		6	-1.979317492	.19877748113
	6	1	.13834266439	2.3164376374
		2	.31380222379	2.4918971968
		3	.19747686414	2.3755718371
		4	-.0012863784	2.1768085946
		5	-.1987774811	1.9793174918
LSD	1	2	-.5635567799	.91447589870
		3	-.6798821396	.79815053905
		4	-.8786453821	.59938729653
		5	-1.076136485	.40189619378
		6	-1.966406490	-.4883738116
	2	1	-.9144758987	.56355677991
		3	-.8553416989	.62269097966
		4	-1.054104941	.42392773714
		5	-1.251596044	.22643663438
		6	-2.141866050	-.6638333710
	3	1	-.7981505391	.67988213956
		2	-.6226909797	.85534169895
		4	-.9377795818	.54025309678
		5	-1.135270685	.34276199403
		6	-2.025540690	-.5475080113
	4	1	-.5993872965	.87864538207
		2	-.4239277371	1.0541049415
		3	-.5402530968	.93777958182
		5	-.9365074421	.54152523655
		6	-1.826777447	-.3487447688
	5	1	-.4018961938	1.0761364848
		2	-.2264366344	1.2515960442
		3	-.3427619940	1.1352706846
		4	-.5415252365	.93650744206
		6	-1.629286345	-.1512536660
	6	1	.48837381158	1.9664064902
		2	.66383337097	2.1418660496
		3	.54750801132	2.0255406899
		4	.34874476880	1.8267774474
		5	.15125366605	1.6292863447

Multiple Comparisons between estrous cycle sampling groups

Dependent Variable		(I) #Treatment	(J) #Treatment	Mean Difference (I-J)	Std. Error	Sig.
AdipoR2	Tukey HSD	1	2	.92045955939	.64184496092	,706
			3	-.0658658003	.64184496092	1,000
			4	-1.109629043	.64184496092	,519
			5	-1.967120146*	.64184496092	,038
			6	-2.747390151*	.64184496092	,001
		2	1	-.9204595594	.64184496092	,706
			3	-.9863253596	.64184496092	,643
			4	-2.030088602*	.64184496092	,029
			5	-2.887579705*	.64184496092	<,001
			6	-3.667849710*	.64184496092	<,001
		3	1	.06586580025	.64184496092	1,000
			2	.98632535964	.64184496092	,643
			4	-1.043763243	.64184496092	,585
			5	-1.901254345*	.64184496092	,049
			6	-2.681524351*	.64184496092	,001
		4	1	1.1096290428	.64184496092	,519
			2	2.030088602*	.64184496092	,029
			3	1.0437632425	.64184496092	,585
			5	-.8574911028	.64184496092	,764
			6	-1.637761108	.64184496092	,128
		5	1	1.967120146*	.64184496092	,038
			2	2.887579705*	.64184496092	<,001
			3	1.901254345*	.64184496092	,049
			4	.85749110275	.64184496092	,764
			6	-.7802700054	.64184496092	,827
		6	1	2.747390151*	.64184496092	,001
			2	3.667849710*	.64184496092	<,001
			3	2.681524351*	.64184496092	,001
			4	1.6377611081	.64184496092	,128
			5	.78027000535	.64184496092	,827
	LSD	1	2	.92045955939	.64184496092	,157
			3	-.0658658003	.64184496092	,919
			4	-1.109629043	.64184496092	,090
			5	-1.967120146*	.64184496092	,003
			6	-2.747390151*	.64184496092	<,001
		2	1	-.9204595594	.64184496092	,157
			3	-.9863253596	.64184496092	,130
			4	-2.030088602*	.64184496092	,003
			5	-2.887579705*	.64184496092	<,001
			6	-3.667849710*	.64184496092	<,001

Multiple Comparisons between estrous cycle sampling groups

Dependent Variable		(I) #Treatment	(J) #Treatment	95% Confidence Interval Lower Bound	95% Confidence Interval Upper Bound
AdipoR2	Tukey HSD	1	2	-.9758583283	2.8167774471
			3	-1.962183688	1.8304520874
			4	-3.005946930	.78668884490
			5	-3.863438033	-.0708022579
			6	-4.643708039	-.8510722632
		2	1	-2.816777447	.97585832828
			3	-2.882643247	.90999252802
			4	-3.926406490	-.1337707145
			5	-4.783897593	-.9912618172
			6	-5.564167598	-1.771531823
		3	1	-1.830452087	1.9621836879
			2	-.9099925280	2.8826432473
			4	-2.940081130	.85255464515
			5	-3.797572233	-.0049364576
			6	-4.577842238	-.7852064630
		4	1	-.7866888449	3.0059469304
			2	.13377071449	3.9264064898
			3	-.8525546452	2.9400811302
			5	-2.753808990	1.0388267849
			6	-3.534078996	.25855677956
		5	1	.07080225786	3.8634380332
			2	.99126181725	4.7838975926
			3	.00493645760	3.7975722329
			4	-1.038826785	2.7538089904
			6	-2.676587893	1.1160478823
		6	1	.85107226321	4.6437080385
			2	1.7715318226	5.5641675979
			3	.78520646296	4.5778422383
			4	-.2585567796	3.5340789958
			5	-1.116047882	2.6765878930
	LSD	1	2	-.3663621090	2.2072812278
			3	-1.352687469	1.2209558681
			4	-2.396450711	.17719262561
			5	-3.253941814	-.6802984771
			6	-4.034211819	-1.460568483
		2	1	-2.207281228	.36636210898
			3	-2.273147028	.30049630873
			4	-3.316910271	-.7432669338
			5	-4.174401373	-1.600758037
			6	-4.954671379	-2.381028042

Multiple Comparisons between estrous cycle sampling groups

Dependent Variable		(I) #Treatment	(J) #Treatment	Mean Difference (I-J)	Std. Error	Sig.
		3	1	.06586580025	.64184496092	,919
			2	.98632535964	.64184496092	,130
			4	-1.043763243	.64184496092	,110
			5	-1.901254345*	.64184496092	,005
			6	-2.681524351*	.64184496092	<,001
		4	1	1.1096290428	.64184496092	,090
			2	2.030088602*	.64184496092	,003
			3	1.0437632425	.64184496092	,110
			5	-.8574911028	.64184496092	,187
			6	-1.637761108*	.64184496092	,014
		5	1	1.967120146*	.64184496092	,003
			2	2.887579705*	.64184496092	<,001
			3	1.901254345*	.64184496092	,005
			4	.85749110275	.64184496092	,187
			6	-.7802700054	.64184496092	,229
		6	1	2.747390151*	.64184496092	<,001
			2	3.667849710*	.64184496092	<,001
			3	2.681524351*	.64184496092	<,001
			4	1.637761108*	.64184496092	,014
			5	.78027000535	.64184496092	,229
RETN	Tukey HSD	1	2	.13545955939	.63330895771	1,000
			3	-.2658658003	.63330895771	,998
			4	.14537095723	.63330895771	1,000
			5	.57787985447	.63330895771	,942
			6	3.227609849*	.63330895771	<,001
		2	1	-.1354595594	.63330895771	1,000
			3	-.4013253596	.63330895771	,988
			4	.00991139784	.63330895771	1,000
			5	.44242029508	.63330895771	,981
			6	3.092150290*	.63330895771	<,001
		3	1	.26586580025	.63330895771	,998
			2	.40132535964	.63330895771	,988
			4	.41123675748	.63330895771	,987
			5	.84374565473	.63330895771	,766
			6	3.493475649*	.63330895771	<,001
		4	1	-.1453709572	.63330895771	1,000
			2	-.0099113978	.63330895771	1,000
			3	-.4112367575	.63330895771	,987
			5	.43250889725	.63330895771	,983
			6	3.082238892*	.63330895771	<,001

Multiple Comparisons between estrous cycle sampling groups

Dependent Variable		(I) #Treatment	(J) #Treatment	95% Confidence Interval	
				Lower Bound	Upper Bound
		3	1	-1.220955868	1.3526874686
			2	-.3004963087	2.2731470280
			4	-2.330584911	.24305842586
			5	-3.188076014	-.6144326769
			6	-3.968346019	-1.394702682
		4	1	-.1771926256	2.3964507111
			2	.74326693379	3.3169102705
			3	-.2430584259	2.3305849109
			5	-2.144312771	.42933056562
			6	-2.924582776	-.3509394397
		5	1	.68029847715	3.2539418139
			2	1.6007580365	4.1744013733
			3	.61443267690	3.1880760137
			4	-.4293305656	2.1443127711
			6	-2.067091674	.50655166302
		6	1	1.4605684825	4.0342118193
			2	2.3810280419	4.9546713786
			3	1.3947026822	3.9683460190
			4	.35093943973	2.9245827765
			5	-.5065516630	2.0670916737
RETN	Tukey HSD	1	2	-1.735638880	2.0065579991
			3	-2.136964240	1.6052326395
			4	-1.725727482	2.0164693970
			5	-1.293218585	2.4489782942
			6	1.3565114094	5.0987082888
		2	1	-2.006557999	1.7356388803
			3	-2.272423799	1.4697730801
			4	-1.861187042	1.8810098376
			5	-1.428678145	2.3135187348
			6	1.2210518500	4.9632487295
		3	1	-1.605232639	2.1369642400
			2	-1.469773080	2.2724237994
			4	-1.459861682	2.2823351972
			5	-1.027352785	2.7148440944
			6	1.6223772096	5.3645740891
		4	1	-2.016469397	1.7257274825
			2	-1.881009838	1.8611870419
			3	-2.282335197	1.4598616822
			5	-1.438589542	2.3036073370
			6	1.2111404522	4.9533373316

Multiple Comparisons between estrous cycle sampling groups

Dependent Variable		(I) #Treatment	(J) #Treatment	Mean Difference (I-J)	Std. Error	Sig.
		5	1	-.5778798545	.63330895771	,942
			2	-.4424202951	.63330895771	,981
			3	-.8437456547	.63330895771	,766
			4	-.4325088972	.63330895771	,983
			6	2.649729995*	.63330895771	,001
		6	1	-3.227609849*	.63330895771	<,001
			2	-3.092150290*	.63330895771	<,001
			3	-3.493475649*	.63330895771	<,001
			4	-3.082238892*	.63330895771	<,001
			5	-2.649729995*	.63330895771	,001
	LSD	1	2	.13545955939	.63330895771	,831
			3	-.2658658003	.63330895771	,676
			4	.14537095723	.63330895771	,819
			5	.57787985447	.63330895771	,366
			6	3.227609849*	.63330895771	<,001
		2	1	-.1354595594	.63330895771	,831
			3	-.4013253596	.63330895771	,529
			4	.00991139784	.63330895771	,988
			5	.44242029508	.63330895771	,488
			6	3.092150290*	.63330895771	<,001
		3	1	.26586580025	.63330895771	,676
			2	.40132535964	.63330895771	,529
			4	.41123675748	.63330895771	,519
			5	.84374565473	.63330895771	,188
			6	3.493475649*	.63330895771	<,001
		4	1	-.1453709572	.63330895771	,819
			2	-.0099113978	.63330895771	,988
			3	-.4112367575	.63330895771	,519
			5	.43250889725	.63330895771	,498
			6	3.082238892*	.63330895771	<,001
		5	1	-.5778798545	.63330895771	,366
			2	-.4424202951	.63330895771	,488
			3	-.8437456547	.63330895771	,188
			4	-.4325088972	.63330895771	,498
			6	2.649729995*	.63330895771	<,001
		6	1	-3.227609849*	.63330895771	<,001
			2	-3.092150290*	.63330895771	<,001
			3	-3.493475649*	.63330895771	<,001
			4	-3.082238892*	.63330895771	<,001
			5	-2.649729995*	.63330895771	<,001

Multiple Comparisons between estrous cycle sampling groups

Dependent Variable		(I) #Treatment	(J) #Treatment	95% Confidence Interval Lower Bound	95% Confidence Interval Upper Bound
		5	1	-2.448978294	1.2932185852
			2	-2.313518735	1.4286781446
			3	-2.714844094	1.0273527850
			4	-2.303607337	1.4385895425
			6	.77863155492	4.5208284344
		6	1	-5.098708289	-1.356511409
			2	-4.963248729	-1.221051850
			3	-5.364574089	-1.622377210
			4	-4.953337332	-1.211140452
			5	-4.520828434	-.7786315549
	LSD	1	2	-1.134248453	1.4051675717
			3	-1.535573813	1.0038422121
			4	-1.124337055	1.4150789696
			5	-.6918281579	1.8475878668
			6	1.9579018368	4.4973178615
		2	1	-1.405167572	1.1342484529
			3	-1.671033372	.86838265269
			4	-1.259796614	1.2796194102
			5	-.8272877173	1.7121283074
			6	1.8224422774	4.3618583021
		3	1	-1.003842212	1.5355738126
			2	-.8683826527	1.6710333720
			4	-.8584712549	1.6809447698
			5	-.4259623576	2.1134536671
			6	2.2237676370	4.7631836617
		4	1	-1.415078970	1.1243370551
			2	-1.279619410	1.2597966145
			3	-1.680944770	.85847125485
			5	-.8371991151	1.7022169096
			6	1.8125308796	4.3519469042
		5	1	-1.847587867	.69182815786
			2	-1.712128307	.82728771725
			3	-2.113453667	.42596235761
			4	-1.702216910	.83719911509
			6	1.3800219823	3.9194380070
		6	1	-4.497317861	-1.957901837
			2	-4.361858302	-1.822442277
			3	-4.763183662	-2.223767637
			4	-4.351946904	-1.812530880
			5	-3.919438007	-1.380021982

*. The mean difference is significant at the 0.05 level.
