# Supplementary material for: Expression of Locally Produced Adipokines and Their Receptors during Different Physiological and Reproductive Stages in the Bovine Corpus Luteum
Source: Animals (Basel). 2023 May 27;13(11):1782. doi: 10.3390/ani13111782 (PMC10251875; doi:10.3390/ani13111782)
Supplement: Supplementary file 1 [file animals-13-01782-s001.zip › Table S2. Multiple comparisons-Pregnancy.pdf]

Multiple Comparisons between pregnancy sampling groups

Dependent Variable		(I) #Group	(J) #Group	Mean Difference (I-J)	Std. Error	Sig.
Vaspin	Tukey HSD	7	8	-1.405815186	1.1364364005	,610
			9	-.5234219961	1.0374197528	,957
			10	-.1129092172	1.0950975168	1,000
		8	7	1.4058151857	1.1364364005	,610
			9	.88239318954	1.0374197528	,830
			10	1.2929059685	1.0950975168	,645
		9	7	.52342199615	1.0374197528	,957
			8	-.8823931895	1.0374197528	,830
			10	.41051277896	.99196301478	,976
		10	7	.11290921719	1.0950975168	1,000
			8	-1.292905968	1.0950975168	,645
			9	-.4105127790	.99196301478	,976
	LSD	7	8	-1.405815186	1.1364364005	,228
			9	-.5234219961	1.0374197528	,618
			10	-.1129092172	1.0950975168	,919
		8	7	1.4058151857	1.1364364005	,228
			9	.88239318954	1.0374197528	,403
			10	1.2929059685	1.0950975168	,249
		9	7	.52342199615	1.0374197528	,618
			8	-.8823931895	1.0374197528	,403
			10	.41051277896	.99196301478	,683
		10	7	.11290921719	1.0950975168	,919
			8	-1.292905968	1.0950975168	,249
			9	-.4105127790	.99196301478	,683
Adipoq	Tukey HSD	7	8	-.8799818524	1.0601652701	,840
			9	-.6478664406	.96779405516	,908
			10	-.6802901696	1.0216008166	,909
		8	7	.87998185235	1.0601652701	,840
			9	.23211541176	.96779405516	,995
			10	.19969168278	1.0216008166	,997
		9	7	.64786644059	.96779405516	,908
			8	-.2321154118	.96779405516	,995
			10	-.0324237290	.92538811414	1,000
		10	7	.68029016957	1.0216008166	,909
			8	-.1996916828	1.0216008166	,997
			9	.03242372898	.92538811414	1,000
	LSD	7	8	-.8799818524	1.0601652701	,415
			9	-.6478664406	.96779405516	,510
			10	-.6802901696	1.0216008166	,512
		8	7	.87998185235	1.0601652701	,415
			9	.23211541176	.96779405516	,812
			10	.19969168278	1.0216008166	,847

Multiple Comparisons between pregnancy sampling groups

Dependent Variable		(I) #Group	(J) #Group	95% Confidence Interval Lower Bound	95% Confidence Interval Upper Bound
Vaspin	Tukey HSD	7	8	-4.540798816	1.7291684448
			9	-3.385257416	2.3384134236
			10	-3.133855033	2.9080365988
		8	7	-1.729168445	4.5407988162
			9	-1.979442230	3.7442286093
			10	-1.728039848	4.3138517845
		9	7	-2.338413424	3.3852574159
			8	-3.744228609	1.9794422302
			10	-2.325925276	3.1469508341
		10	7	-2.908036599	3.1338550332
			8	-4.313851785	1.7280398475
			9	-3.146950834	2.3259252762
	LSD	7	8	-3.751304638	.93967426659
			9	-2.664551132	1.6177071395
			10	-2.373079407	2.1472609727
		8	7	-.9396742666	3.7513046380
			9	-1.258735946	3.0235223252
			10	-.9672642214	3.5530761584
		9	7	-1.617707140	2.6645511318
			8	-3.023522325	1.2587359461
			10	-1.636798260	2.4578238183
		10	7	-2.147260973	2.3730794070
			8	-3.553076158	.96726422136
			9	-2.457823818	1.6367982604
Adipoq	Tukey HSD	7	8	-3.804563260	2.0445995549
			9	-3.317631787	2.0218989061
			10	-3.498487330	2.1379069910
		8	7	-2.044599555	3.8045632596
			9	-2.437649935	2.9018807585
			10	-2.618505478	3.0178888433
		9	7	-2.021898906	3.3176317873
			8	-2.901880758	2.4376499349
			10	-2.585207667	2.5203602091
		10	7	-2.137906991	3.4984873301
			8	-3.017888843	2.6185054778
			9	-2.520360209	2.5852076670
	LSD	7	8	-3.068055428	1.3080917236
			9	-2.645295199	1.3495623178
			10	-2.788770625	1.4281902863
		8	7	-1.308091724	3.0680554283
			9	-1.765313347	2.2295441702
			10	-1.908788773	2.3081721387

Multiple Comparisons between pregnancy sampling groups

Dependent Variable		(I) #Group	(J) #Group	Mean Difference (I-J)	Std. Error	Sig.
		9	7	.64786644059	.96779405516	,510
			8	-.2321154118	.96779405516	,812
			10	-.0324237290	.92538811414	,972
		10	7	.68029016957	1.0216008166	,512
			8	-.1996916828	1.0216008166	,847
			9	.03242372898	.92538811414	,972
HSPA5	Tukey HSD	7	8	-.0566485190	.25925022921	,996
			9	.8365780039*	.23666199763	,009
			10	.07613840186	.24981977182	,990
		8	7	.05664851902	.25925022921	,996
			9	.8932265229*	.23666199763	,005
			10	.13278692088	.24981977182	,951
		9	7	-.8365780039*	.23666199763	,009
			8	-.8932265229*	.23666199763	,005
			10	-.7604396020*	.22629215225	,013
		10	7	-.0761384019	.24981977182	,990
			8	-.1327869209	.24981977182	,951
			9	.7604396020*	.22629215225	,013
	LSD	7	8	-.0566485190	.25925022921	,829
			9	.8365780039*	.23666199763	,002
			10	.07613840186	.24981977182	,763
		8	7	.05664851902	.25925022921	,829
			9	.8932265229*	.23666199763	<,001
			10	.13278692088	.24981977182	,600
		9	7	-.8365780039*	.23666199763	,002
			8	-.8932265229*	.23666199763	<,001
			10	-.7604396020*	.22629215225	,003
		10	7	-.0761384019	.24981977182	,763
			8	-.1327869209	.24981977182	,600
			9	.7604396020*	.22629215225	,003
AdipoR1	Tukey HSD	7	8	.01001814765	.16637026839	1,000
			9	.23657800385	.15187458149	,421
			10	.07851935424	.16031840208	,961
		8	7	-.0100181476	.16637026839	1,000
			9	.22655985620	.15187458149	,458
			10	.06850120659	.16031840208	,973
		9	7	-.2365780039	.15187458149	,421
			8	-.2265598562	.15187458149	,458
			10	-.1580586496	.14521987586	,700

Multiple Comparisons between pregnancy sampling groups

Dependent Variable		(I) #Group	(J) #Group	95% Confidence Interval Lower Bound	Upper Bound
		9	7	-1.349562318	2.6452951990
			8	-2.229544170	1.7653133466
			10	-1.942330927	1.8774834687
		10	7	-1.428190286	2.7887706255
			8	-2.308172139	1.9087887731
			9	-1.877483469	1.9423309267
HSPA5	Tukey HSD	7	8	-.7718185228	.65852148475
			9	.18372009799	1.4894359097
			10	-.6130166577	.76529346143
		8	7	-.6585214847	.77181852279
			9	.24036861701	1.5460844287
			10	-.5563681387	.82194198044
		9	7	-1.489435910	-.1837200980
			8	-1.546084429	-.2403686170
			10	-1.384691160	-.1361880445
		10	7	-.7652934614	.61301665771
			8	-.8219419804	.55636813869
			9	.13618804446	1.3846911595
	LSD	7	8	-.5917146942	.47841765616
			9	.34813164735	1.3250243604
			10	-.4394642659	.59174106959
		8	7	-.4784176562	.59171469419
			9	.40478016637	1.3816728794
			10	-.3828157469	.64838958861
		9	7	-1.325024360	-.3481316473
			8	-1.381672879	-.4047801664
			10	-1.227483650	-.2933955545
		10	7	-.5917410696	.43946426587
			8	-.6483895886	.38281574685
			9	.29339555446	1.2274836495
AdipoR1	Tukey HSD	7	8	-.4489323645	.46896865979
			9	-.1823845766	.65554058432
			10	-.3637364253	.52077513380
		8	7	-.4689686598	.44893236450
			9	-.1924027243	.64552243667
			10	-.3737545730	.51075698615
		9	7	-.6555405843	.18238457662
			8	-.6455224367	.19240272426
			10	-.5586634995	.24254620032

Multiple Comparisons between pregnancy sampling groups

Dependent Variable		(I) #Group	(J) #Group	Mean Difference (I-J)	Std. Error	Sig.
		10	7	-.0785193542	.16031840208	,961
			8	-.0685012066	.16031840208	,973
			9	.15805864961	.14521987586	,700
	LSD	7	8	.01001814765	.16637026839	,952
			9	.23657800385	.15187458149	,132
			10	.07851935424	.16031840208	,629
		8	7	-.0100181476	.16637026839	,952
			9	.22655985620	.15187458149	,149
			10	.06850120659	.16031840208	,673
		9	7	-.2365780039	.15187458149	,132
			8	-.2265598562	.15187458149	,149
			10	-.1580586496	.14521987586	,287
		10	7	-.0785193542	.16031840208	,629
			8	-.0685012066	.16031840208	,673
			9	.15805864961	.14521987586	,287
AdipoR2	Tukey HSD	7	8	-.9649818524	.66761372115	,485
			9	-.9217553295	.60944515796	,446
			10	-.7274330267	.64332867898	,675
		8	7	.96498185235	.66761372115	,485
			9	.04322652287	.60944515796	1,000
			10	.23754882564	.64332867898	,982
		9	7	.92175532948	.60944515796	,446
			8	-.0432265229	.60944515796	1,000
			10	.19432230277	.58274103089	,987
		10	7	.72743302671	.64332867898	,675
			8	-.2375488256	.64332867898	,982
			9	-.1943223028	.58274103089	,987
	LSD	7	8	-.9649818524	.66761372115	,161
			9	-.9217553295	.60944515796	,143
			10	-.7274330267	.64332867898	,269
		8	7	.96498185235	.66761372115	,161
			9	.04322652287	.60944515796	,944
			10	.23754882564	.64332867898	,715
		9	7	.92175532948	.60944515796	,143
			8	-.0432265229	.60944515796	,944
			10	.19432230277	.58274103089	,742
		10	7	.72743302671	.64332867898	,269
			8	-.2375488256	.64332867898	,715
			9	-.1943223028	.58274103089	,742

Multiple Comparisons between pregnancy sampling groups

Dependent Variable		(I) #Group	(J) #Group	95% Confidence Interval Lower Bound	Upper Bound
		10	7	-.5207751338	.36373642532
			8	-.5107569862	.37375457297
			9	-.2425462003	.55866349955
	LSD	7	8	-.3333532100	.35338950528
			9	-.0768757264	.55003173414
			10	-.2523615652	.40940027370
		8	7	-.3533895053	.33335320998
			9	-.0868938741	.54001358650
			10	-.2623797129	.39938212606
		9	7	-.5500317341	.07687572644
			8	-.5400135865	.08689387409
			10	-.4577777425	.14166044330
		10	7	-.4094002737	.25236156522
			8	-.3993821261	.26237971287
			9	-.1416604433	.45777774253
AdipoR2	Tukey HSD	7	8	-2.806667042	.87670333698
			9	-2.602976200	.75946554055
			10	-2.502125281	1.0472592278
		8	7	-.8767033370	2.8066670417
			9	-1.637994347	1.7244473929
			10	-1.537143429	2.0122410802
		9	7	-.7594655406	2.6029761995
			8	-1.724447393	1.6379943472
			10	-1.413232323	1.8018769287
		10	7	-1.047259228	2.5021252812
			8	-2.012241080	1.5371434289
			9	-1.801876929	1.4132323232
	LSD	7	8	-2.342868851	.41290514646
			9	-2.179588314	.33607765542
			10	-2.055198162	.60033210849
		8	7	-.4129051465	2.3428688512
			9	-1.214606462	1.3010595078
			10	-1.090216310	1.5653139608
		9	7	-.3360776554	2.1795883144
			8	-1.301059508	1.2146064620
			10	-1.008396073	1.3970406782
		10	7	-.6003321085	2.0551981619
			8	-1.565313961	1.0902163096
			9	-1.397040678	1.0083960727

Multiple Comparisons between pregnancy sampling groups

Dependent Variable		(I) #Group	(J) #Group	Mean Difference (I-J)	Std. Error	Sig.
RETN	Tukey HSD	7	8	-3.539981852*	.57415658400	<,001
			9	-4.099533107*	.52413085433	<,001
			10	-4.356004455*	.55327112821	<,001
		8	7	3.539981852*	.57415658400	<,001
			9	-.5595512549	.52413085433	,712
			10	-.8160226029	.55327112821	,467
		9	7	4.099533107*	.52413085433	<,001
			8	.55955125491	.52413085433	,712
			10	-.2564713480	.50116495370	,955
		10	7	4.356004455*	.55327112821	<,001
			8	.81602260293	.55327112821	,467
			9	.25647134803	.50116495370	,955
	LSD	7	8	-3.539981852*	.57415658400	<,001
			9	-4.099533107*	.52413085433	<,001
			10	-4.356004455*	.55327112821	<,001
		8	7	3.539981852*	.57415658400	<,001
			9	-.5595512549	.52413085433	,296
			10	-.8160226029	.55327112821	,153
		9	7	4.099533107*	.52413085433	<,001
			8	.55955125491	.52413085433	,296
			10	-.2564713480	.50116495370	,614
		10	7	4.356004455*	.55327112821	<,001
			8	.81602260293	.55327112821	,153
			9	.25647134803	.50116495370	,614
NAMPT	Tukey HSD	7	8	.07668481431	.47139422236	,998
			9	.05602244830	.43032208178	,999
			10	-.5036235029	.45424683877	,688
		8	7	-.0766848143	.47139422236	,998
			9	-.0206623660	.43032208178	1,000
			10	-.5803083172	.45424683877	,585
		9	7	-.0560224483	.43032208178	,999
			8	.02066236602	.43032208178	1,000
			10	-.5596459512	.41146661069	,535
		10	7	.50362350290	.45424683877	,688
			8	.58030831722	.45424683877	,585
			9	.55964595120	.41146661069	,535
	LSD	7	8	.07668481431	.47139422236	,872
			9	.05602244830	.43032208178	,898
			10	-.5036235029	.45424683877	,279

Multiple Comparisons between pregnancy sampling groups

Dependent Variable		(I) #Group	(J) #Group	95% Confidence Interval Lower Bound	Upper Bound
RETN	Tukey HSD	7	8	-5.123855346	-1.956108358
			9	-5.545405175	-2.653661039
			10	-5.882263144	-2.829745767
		8	7	1.9561083584	5.1238553463
			9	-2.005423323	.88632081319
			10	-2.342281291	.71023608563
		9	7	2.6536610392	5.5454051754
			8	-.8863208132	2.0054233230
			10	-1.638989477	1.1260467810
		10	7	2.8297457667	5.8822631438
			8	-.7102360856	2.3422812915
			9	-1.126046781	1.6389894770
	LSD	7	8	-4.724982800	-2.354980904
			9	-5.181286024	-3.017780191
			10	-5.497899941	-3.214108970
		8	7	2.3549809045	4.7249828002
			9	-1.641304171	.52220166145
			10	-1.957918089	.32587288278
		9	7	3.0177801909	5.1812860236
			8	-.5222016615	1.6413041713
			10	-1.290824975	.77788227906
		10	7	3.2141089696	5.4978999410
			8	-.3258728828	1.9579180886
			9	-.7778822791	1.2908249751
NAMPT	Tukey HSD	7	8	-1.223707509	1.3770771372
			9	-1.131067900	1.2431127964
			10	-1.756712900	.74946589408
		8	7	-1.377077137	1.2237075086
			9	-1.207752714	1.1664279821
			10	-1.833397714	.67278107976
		9	7	-1.243112796	1.1310678998
			8	-1.166427982	1.2077527141
			10	-1.694721428	.57542952555
		10	7	-.7494658941	1.7567128999
			8	-.6727810798	1.8333977142
			9	-.5754295255	1.6947214279
	LSD	7	8	-.8962250432	1.0495946718
			9	-.8321186773	.94416357391
			10	-1.441142900	.43389589425

Multiple Comparisons between pregnancy sampling groups

Dependent Variable	(I) #Group	(J) #Group	Mean Difference (I-J)	Std. Error	Sig.
	8	7	-.0766848143	.47139422236	,872
		9	-.0206623660	.43032208178	,962
		10	-.5803083172	.45424683877	,214
	9	7	-.0560224483	.43032208178	,898
		8	.02066236602	.43032208178	,962
		10	-.5596459512	.41146661069	,186
	10	7	.50362350290	.45424683877	,279
		8	.58030831722	.45424683877	,214
		9	.55964595120	.41146661069	,186

Multiple Comparisons between pregnancy sampling groups

Dependent Variable	(I) #Group	(J) #Group	95% Confidence Interval	
			Lower Bound	Upper Bound
	8	7	-1.049594672	.89622504318
		9	-.9088034916	.86747875960
		10	-1.517827714	.35721107994
	9	7	-.9441635739	.83211867732
		8	-.8674787596	.90880349163
		10	-1.408871297	.28957939477
	10	7	-.4338958943	1.4411429001
		8	-.3572110799	1.5178277144
		9	-.2895793948	1.4088712972

*. The mean difference is significant at the 0.05 level.
